# Establishing an AI-based artifact correction system for intrarenal pressure monitoring using the LithoVue™ Elite ureteroscope: an EAU endourology and AUSET collaboration: Author list

**DOI:** 10.1007/s00345-025-06057-7

**Published:** 2025-11-10

**Authors:** Takahiro Yanase, Shuzo Hamamoto, Rei Unno, Steffi Kar Kei Yuen, Vineet Gauhar, Bhaskar K. Somani, Olivier Traxer, Yuya Sasaki, Ryosuke Chaya, Atsushi Okada, Kazumi Taguchi, Takahiro Yasui

**Affiliations:** 1 Department of Nephro-urology, Nagoya City University Graduate School of Medical Sciences, Nagoya, 467-8601 Japan; 2 SH Ho Urology Centre, Department of Surgery, The Chinese University of Hong Kong, Hong Kong, 999077 China; 3 Ng Teng Fong General Hospital, (NUHS), Singapore, 609606 Singapore; 4 Asian Institute of Nephrourology (AINU), Hyderabad, 500082 India; 5 Department of Urology, University Hospital Southampton, NHS Trust, Southampton, UK; 6 Department of Urology, Sorbonne University, AP-HP, Hôpital Tenon, GRC n°20 Lithiase Renale, Paris, F-75020 France; 7 Graduate School of Information Science and Technology, The University of Osaka, Osaka, 565-0871 Japan; 8 Department of Urology, University of Alabama at Birmingham, Birmingham, AL 35294 USA

**Keywords:** Intrarenal pressure, LithoVue Elite, Kidney stone, Artificial intelligence, Machine learning, Retrograde intrarenal surgery

## Abstract

**Purpose:**

Intrarenal pressure (IRP) management during endoscopic surgery for urolithiasis is critical to minimize postoperative pain and infectious complications. However, pressure sensors at the ureteroscope tip often register various artifacts when contacting the pelvicalyceal or ureteral wall, leading to significant deviations from true IRP values. This study aimed to develop and validate a machine learning model to detect and remove artifacts from IRP data accurately.

**Materials and methods:**

We analyzed 27 retrograde intrarenal surgeries performed using the Boston Scientific^®^ LithoVue™ Elite system across academic institutions in Japan and Hong Kong. 32 waveform features were identified and three tree-based machine learning models—Random Forest, XGBoost, and LightGBM—were trained for automated artifact detection. Endpoints included agreement with ground-truth labels and comparison of time efficiency between artificial intelligence (AI)-based and manual annotations.

**Results:**

The best-performing model achieved an overall agreement of 93.6% and an area under the receiver operating characteristic curve of 0.95. Each case included 94 artifacts, contributing 258 s per surgery. Artifacts accounted for 31% of the time > 30 mmHg and 72% of the time > 100 mmHg. Without correction, peak IRP was overestimated by 184 mmHg (median, 257 vs. 73 mmHg). False negatives > 60 mmHg had a median 0.0 s per case. AI-based IRP annotation saved 99.95% of the time compared to manual review (1.8 s vs. 56.6 min per case).

**Conclusions:**

The model successfully achieved high-precision artifact removal from IRP data. This system may serve as a standardized process for accurate IRP analysis during endoscopic surgery.

**Supplementary Information:**

The online version contains supplementary material available at 10.1007/s00345-025-06057-7.

## Introduction

Retrograde intrarenal surgery (RIRS) has become the cornerstone of urolithiasis treatment, supported by advances in flexible ureteroscopy and laser technology [1]. Despite its minimally invasive nature, postoperative infectious complications remain a concern, with sepsis reported in approximately 5% of RIRS cases [2]. The risk factors for post-RIRS sepsis include older age, positive preoperative urine culture, diabetes mellitus, and elevated intraoperative intrarenal pressure (IRP) [3, 4]. The IRP is a modifiable procedural variable that requires careful monitoring and control. Modern flexible ureteroscopy involves a delicate balance among the quadrifecta of suction, irrigation, intrarenal temperature, and IRP [5]. This makes the precision of IRP monitoring integral.

With the introduction of pressure-sensor-equipped flexible ureteroscopes, such as the LithoVue™ Elite (LVE; Boston Scientific, Marlborough, MA, USA), real-time IRP monitoring has become feasible [6]. However, direct pressure measurements at the ureteroscope tip are prone to frequent artifactual spikes caused by physical contact between the pressure sensor and the pelvicalyceal or ureteral wall, potentially leading to significant distortion of true IRP readings. This is even more pronounced based on the anatomy of the pelvicalyceal system, stone location, and the equipment used. The analysis of IRP data can be confusing and time-consuming, often necessitating a correlation with endoscopic views.

To date, no study has systematically quantified the prevalence of such artifacts or developed robust correction methodologies. In this study, we aimed to (1) quantify the frequency and magnitude of artifacts within IRP data collected during RIRS, and (2) develop and validate an artificial intelligence (AI)-based model to detect and correct such artifacts.

## Materials and methods

### Study design and patient selection

Prospectively collected data of patients who underwent RIRS using the LVE system with continuous IRP monitoring or endoscopic combined intrarenal surgery (ECIRS) for renal stones between August 2023 and January 2025 at Nagoya City University Hospital, its affiliated institution in Japan, and the Chinese University of Hong Kong were retrospectively analyzed. Patients were excluded if they were < 18 years of age, unable to provide informed consent, had bleeding diatheses, severe ureteral strictures, active urinary tract infection, urinary diversion, pregnancy, or American Society of Anesthesiologists Physical Status classification of III or higher.

### Ethical considerations

This study was approved by the Institutional Review Board of the Nagoya City University Hospital (approval number: 60-19-0044) and the Clinical Research Ethics Committee of the Chinese University of Hong Kong (CREC Ref. No.: 2021.684). Written informed consent was obtained from all patients before their participation. All data were anonymized to ensure patient confidentiality in accordance with the Declaration of Helsinki.

### Surgical procedures

All procedures followed standardized RIRS or ECIRS protocols. ECIRS was performed with the patient in either the Galdakao-modified supine Valdivia or prone split-leg position to allow simultaneous antegrade and retrograde access. Percutaneous access was established under ultrasound guidance, followed by tract dilation to 17.5 or 19.5 Fr. A flexible ureteroscope (LVE) was introduced retrogradely and laser lithotripsy was performed using a Holmium: YAG laser (Cyber Ho^®^, Quanta System, Milan, Italy) in either dusting or fragmentation mode. For percutaneous nephrolithotomy, the Swiss LithoClast^®^ Master was employed. A ureteral access sheath (11/13 or 12/14 Fr) was used. A 6 Fr double-J stent was placed preoperatively at the discretion of the operating urologist. Irrigation was provided either with an automated endoscopic irrigation system (UROMAT^®^, Karl Storz, Germany) set to deliver a pressure of 90 mmHg or with a manual foot pump (Peditrol^®^).

### IRP monitoring

The LVE is a 9.5 Fr single-use flexible ureteroscope with a 7.7 Fr distal tip housing a lateral pressure sensor capable of real-time IRP monitoring at 4 Hz [7]. Calibration to zero was performed by immersing the ureteroscope tip in saline prior to clinical use. IRP measurements began once the ureteral access sheath was in place and the ureteroscope tip was positioned in the renal pelvis. Monitoring was continued throughout the procedure until the final ureteroscopic inspection and scope withdrawal. Based on prior evidence of pyelovenous backflow risk, the safe IRP threshold was defined as 30 mmHg [8].

### Artifact labeling and AI-based correction model

To develop a robust correction system for IRP artifacts, synchronized endoscopic video and IRP waveform data were retrospectively reviewed to manually label transient spike artifacts primarily caused by ureteroscopic contact with the pelvicalyceal or ureteral walls. Two experienced urologists performed labeling in consensus. To compare cumulative IRP exposure between raw measurements and the ground truth, IRP values were stratified into clinical ranges, and statistical differences were evaluated using the Wilcoxon signed-rank test (*p* < 0.05).

Of the 27 IRP records, 20 were used for model training and 7 for external testing. The external test set consisted of cases from the Chinese University of Hong Kong and affiliated institutions that were excluded from the training phase to enable independent validation. All analyses were performed using Python (v.3.10.14).

To prevent data leakage and ensure robust evaluations, the training data were split on a case-wise basis using the GroupShuffleSplit method (Scikit-learn v.1.5.1), with 80% of the cases allocated for model training and 20% for internal validation. Hyperparameters were optimized using 5-fold stratified cross-validation within the training set using StratifiedKFold with a fixed random seed (42), with the objective of maximizing the mean F1-score across folds. The final model was retrained on the entire training set using the best-performing parameters. Thirty-two waveform-derived features were extracted to characterize the morphological, temporal, and frequency aspects of IRP signals, including derivatives, peak shape, short-term energy, and frequency-domain metrics (see Supplementary Table S1 for details).

To address the class imbalance between artifactual and non-artifactual segments, a synthetic minority oversampling technique (SMOTE; imbalanced-learn v.0.11.0) was applied, followed by the additional oversampling of high-pressure artifact segments. Three tree-based classifiers were implemented: Random Forest (scikit-learn v.1.5.1), Light Gradient Boosting Machine (LightGBM v.4.5.0), and eXtreme Gradient Boosting (XGBoost v.2.1.2). Their hyperparameters (number of trees, depth, learning rate, and subsampling ratios) were optimized using Optuna (v.3.4.0) to maximize the F1-score. To enhance temporal consistency and minimize false predictions, a multi-stage post-processing pipeline was developed and optimized using Optuna, with particular emphasis on minimizing false negatives in high-pressure segments (≥ 30 mmHg).

The predictive performance of the model was evaluated using accuracy, recall, F1-score, and area under the receiver operating characteristic curve (AUC). Accuracy was evaluated for the entire dataset as well as specifically for high-pressure segments. Additionally, SHapley Additive exPlanations (SHAP) analysis was conducted to assess feature importance and enhance model interpretability.

Finally, the time efficiency was assessed by comparing the duration required for AI-based detection and manual expert labeling. For the AI system, the processing time was defined as the time elapsed from inputting the test data to generating the predicted labels, excluding the model loading and initialization time. The manual labeling time was recorded as the total duration required by the experienced urologists to complete artifact labeling for each case.

## Results

### Scope of artifacts

 In total, 518,505 IRP waveform data points were analyzed from 27 surgical cases, with a median of 16,173 samples per case (interquartile range [IQR]: 10,494–28,983). The LVE system was used for a median of 69.3 min per procedure (IQR: 59–102), corresponding to a total cumulative recording time of 37.0 h across all cases. Before correction, each case exhibited a median of 94 (IQR: 60–166) artifactual spikes, amounting to 258 (IQR: 163–566) s, corresponding to approximately 6.2% of the total operative time. When limited to segments where IRP > 30 mmHg, there was a median of 75 (IQR: 34–111) artifactual spikes, amounting to 156 (IQR: 58–180) s, corresponding to 31% of the high-pressure duration (median 502 s). For pressures > 100 mmHg, a median of 12 (IQR: 2–20) artifacts accounted for 13 (IQR: 3–24) s, which constituted 72% of the extreme-pressure period (median 18 s) (Table 1).

### Model performance

**Fig. 1:**
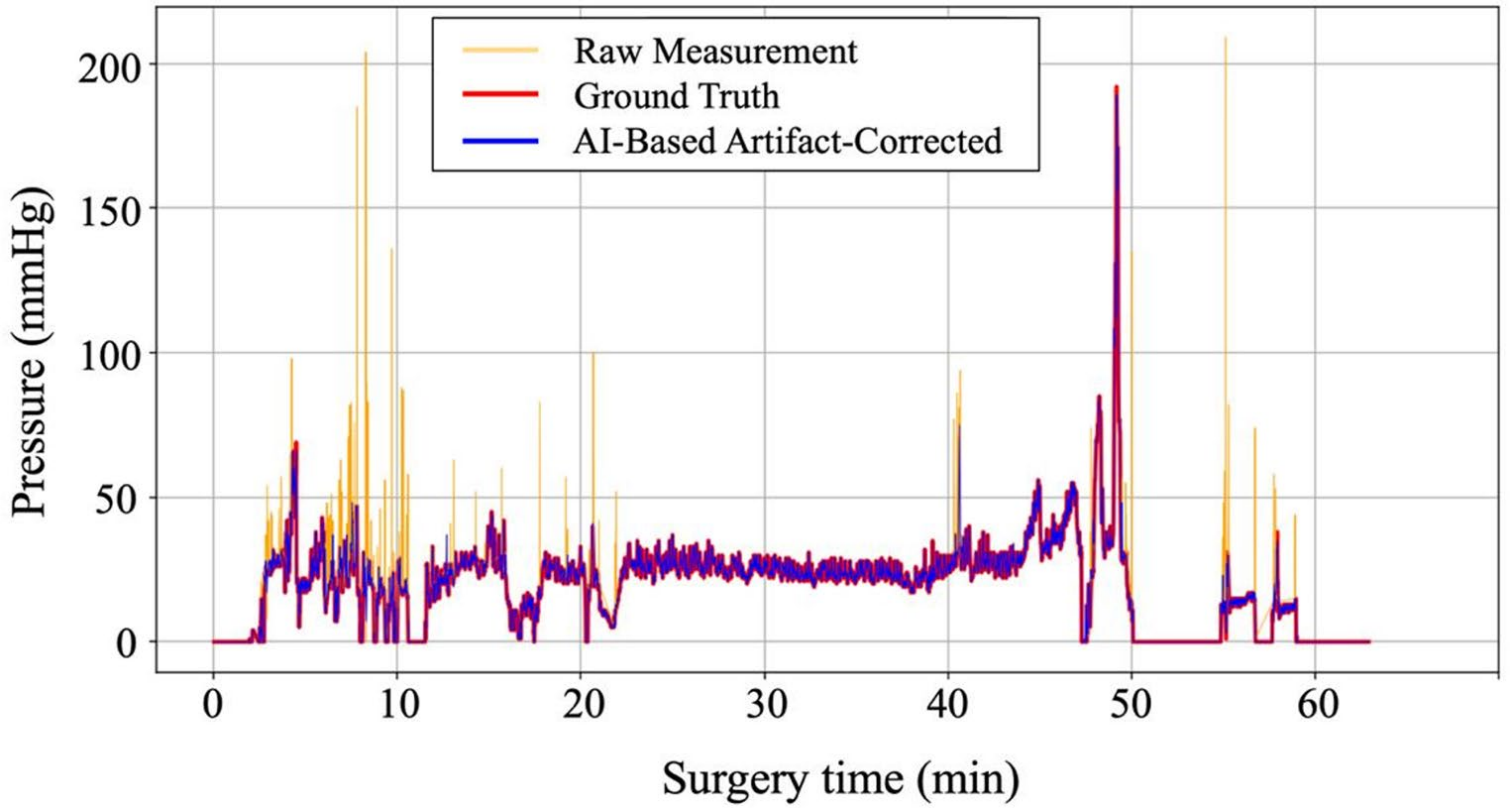
AI-based Artifact Correction of IRP Waveform (example case). Orange line: raw IRP measurements with spike artifacts. Blue line: AI-corrected output. Ground-truth (red) and AI-corrected (blue) traces aligned closely, confirming precise artifact exclusion.

: On the entire test set, LightGBM outperformed the other classifiers, achieving agreement of 93.6% (95% confidence interval [CI]: 93.4–93.7), recall of 0.70 (0.70–0.71), specificity of 0.95 (0.95–0.95) and AUC of 0.95 (0.95–0.95) (Supplementary Table S2). At IRP ≥ 30 mmHg, the model achieved agreement of 82.7% (82.1–83.3), recall of 0.87 (0.86–0.88), specificity of 0.81 (0.81–0.82) and AUC of 0.97 (0.97–0.97) (Supplementary Table S3). An illustrative example of artifact correction is shown in Fig. 1.

**Table 1:** Comparison of IRP parameters before and after artifact correction (*n* = 27)

	Raw data	Ground truth	*p* value*
Duration within IRP Range (s) ^a)^			< 0.05
20–29	304 [170, 701]	230 [70, 611]	
30–39	130 [44, 319]	102 [6, 212]	
40–49	67 [28, 188]	38 [2, 112]	
50–59	46 [17, 108]	11 [0, 83]	
60–79	46 [20, 141]	12 [0, 75]	
80–99	14 [4, 35]	0 [0, 7]	
≥100	15 [6, 25]	0 [0, 5]	
Maximum IRP (mmHg) ^a)^	257 [208, 300]	73 [50, 113]	< 0.05
Mean IRP (mmHg) ^a)^	13 [9, 19]	11 [5, 18]	< 0.05
Duration of IRP > 30 mmHg (s) ^a)^	502 [259, 781]	114 [24, 528]	< 0.05

a) Median [25%, 75% interquartile range]

*P values were calculated using the Wilcoxon signed-rank test.

*IRP* intrarenal pressure.

### Effect on peak pressure metrics

The median maximum IRP decreased from 257 mmHg (IQR: 208–300) in the uncorrected signal to 73 mmHg (IQR: 50–113 mmHg) following manual annotation, a reduction of 184 mmHg, corresponding to an approximately 252% overestimation. The AI-corrected values closely replicated the ground truth with a median difference of < 1 mmHg.

**Fig. 2:**
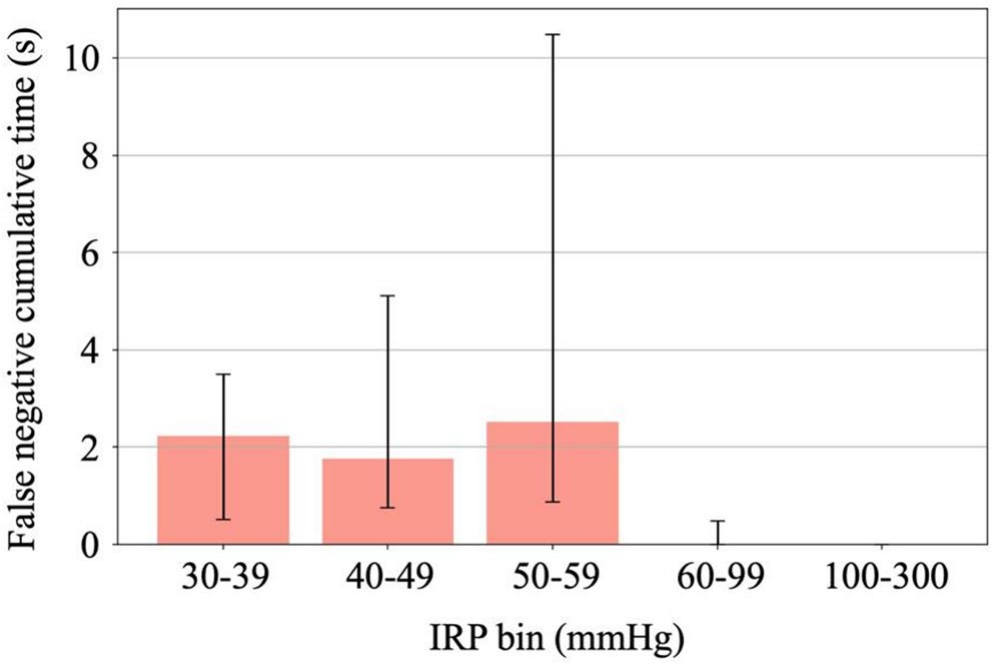
Cumulative Duration of Missed Artifacts (False Negatives) by IRP Bin

Error analysis: 85% of the missed artifacts occurred at an IRP < 30 mmHg. In segments > 60 mmHg, missed artifacts were minimal—limited to a median duration of 0.0 s (IQR: 0–0.1 s) per case—and no false negatives were observed > 100 mmHg (Fig. 2).

Bar heights represent the median cumulative seconds per case of undetected artifact spikes (false negatives) within each intrarenal pressure (IRP) range and whiskers denote the interquartile range. Even at higher pressures (60–99 mmHg), the median missed time remained minimal, and no false negatives occurred > 100 mmHg.

### Feature importance (SHAP) analysis

The top seven features accounted for more than 50% of the model’s predictive output (see Supplementary Figure S1). The most influential features were Fast Fourier Transform Dominant Frequency (13.5%), Peak Count in the last 120 points (7.8%), Absolute Pressure (6.9%), Edge Position (maximum in the last 10 points) (6.8%), Duration Above Threshold (> 30 mmHg) (6.3%), Absolute Curvature (maximum in the last 10 points) (6.0%), and Peak Area (rolling sum over 10 points) (5.7%).

### Time efficiency

The AI system completed artifact detection for each case in a median of 1.8 s. In contrast, manual expert annotation required a median of 56.6 min per case (*p* < 0.001), corresponding to an approximately 1,900-fold reduction in processing time.

## Discussion

The lack of standardized artifact correction in previous IRP studies likely contributes to the wide variability in reported peak pressures and exposure times [9]. For example, earlier clinical studies using LVE or pressure sensor guidewires reported extremely high maximum IRP values, sometimes exceeding 300 mmHg [10]. However, it remains unclear whether these values represent true physiological elevations or are attributable to unrecognized artifacts, highlighting the need for further verification and correction of artifacts in IRP measurements. To address this, this study systematically quantified the prevalence and impact of artifacts in IRP measurements and demonstrated the utility of an AI-based artifact correction system. Notably, our model achieved high overall agreement (93.6%) and AUC (0.95), and in clinically relevant high-pressure segments (IRP ≥ 30 mmHg), agreement was 82.7% and AUC was 0.97. The median maximum IRP was overestimated by 184 mmHg in uncorrected data (257 vs. 73 mmHg), and artifactual spikes occurred 31% of the time > 30 mmHg and 72% >100 mmHg. After AI-based correction, maximum IRP values closely matched the ground truth. Error analysis revealed almost all false negatives occurred at low pressures, with only 0.0 s (IQR: 0–0.1 s) missed > 60 mmHg, and no undetected events > 100 mmHg. These results underscore the need for robust IRP analysis to optimize endoscopic outcomes.

As IRP monitoring and reporting using various devices has become a reality in flexible ureteroscopy [11], accurate assessment of IRP is essential to ensure intraoperative safety and prevent complications, particularly when devices such as flexible and navigable suction ureteral access sheaths (FANS) are used, which may elevate IRP if outflow is not balanced [12]. However, sensor-equipped ureteroscopes are highly susceptible to artifactual spikes from transient contact with the ureteral or pelvic wall, debris, or stone fragments. In contrast, true IRP elevations from irrigation pressure or outflow obstruction must be distinguished from artifacts. Such artifacts can substantially overestimate peak pressures and high-pressure durations, leading to potential misinterpretation of clinical risks and hindering comparability across studies.

Since the launch of the LVE and other pressure-sensor-equipped devices, the management of robust IRP datasets has weighed investigators’ skills owing to the lack of relevant evidence, including guidelines. The SHAP analysis confirmed that the model’s predictions were driven by intuitive features reflecting spike steepness and local variability, aligned with expert interpretation. The system’s time efficiency is also noteworthy, completing artifact detection in 1.8 s per case, compared to 56.6 min for manual annotation, facilitating large-scale or multi-institutional studies. Given the complexity and time required for manual annotation, this automated approach enables efficient processing of large datasets and greatly reduces the burden on clinicians and researchers, preserving valuable effort for other critical tasks.

Although our model was designed for retrospective analysis rather than real-time intraoperative support, it enables standardized, objective evaluation of pressure exposure. This capability is expected to improve the accuracy and clinical relevance of IRP research, facilitate meta-analyses, and help establish evidence-based safety thresholds for endourology. Although in vitro and clinical studies have already yielded comparable and reliable data from current novel non-invasive IRP monitoring devices, including IRP-sensing flexible ureteroscopes and IRP-sensing FANS [7, 13], our model’s application to IRP datasets from various IRP sensing devices can further quantify, adjust, and standardize the quality of IRP data recorded. Future directions may include automatic artifact removal directly integrated into pressure-sensing scopes for robust reporting. However, our findings may be specific to LVE-derived waveforms, limiting reproducibility with other scopes.

## Conclusions

IRP data obtained using the LVE frequently contained diverse and high-frequency artifacts, primarily owing to physical contact with the pressure sensor. The AI-based artifact correction system developed in this study demonstrates high accuracy in identifying and removing such artifacts. This system offers a promising foundation for standardizing IRP analysis and may contribute to a more efficient and reliable interpretation of IRP in clinical and research settings.

## Supplementary Information

Below is the link to the electronic supplementary material.

Supplementary Material 1

## Data Availability

The data and codes presented in this study are not publicly available because of intellectual property considerations related to ongoing patent applications. Access requests will be considered on a case-by-case basis and will require approval from the corresponding author and the institutional ethics committee.

## Abbreviations

AI: Artificial intelligence
AUC: Area under the curve
CI: Confidence interval
ECIRS: Endoscopic combined intrarenal surgery
IQR: Interquartile range
IRP: Intrarenal pressure
LVE: LithoVue™ Elite
LightGBM: Light gradient boosting machine
RIRS: Retrograde intrarenal surgery
ROC: Receiver operating characteristic
SHAP: SHapley additive exPlanations
SMOTE: Synthetic minority over-sampling technique
XGBoost: eXtreme gradient boosting
