# Supplementary material for: Establishing an AI-based artifact correction system for intrarenal pressure monitoring using the LithoVue™ Elite ureteroscope: an EAU endourology and AUSET collaboration: Author list
Source: World J Urol. 2025 Nov 10;43(1):683. doi: 10.1007/s00345-025-06057-7 (PMC12602662; doi:10.1007/s00345-025-06057-7)
Supplement: Supplementary file 1 — Supplementary Material 1 [file 345_2025_6057_MOESM1_ESM.docx]

**Supplementary Table S1. List of Extracted Features for Machine Learning Model**

| **Category** | **Feature Name** |
| --- | --- |
| **1. Basic / Raw** | Pressure |
|  | Duration above threshold |
| **2. Waveform Derivatives** | First derivative (Velocity) |
|  | Second derivative (Acceleration) |
|  | Third derivative (Jerk) |
| **3. Local Curvature & Edge** | Edge position (max, min, last 10) |
|  | Absolute curvature (max 10) |
| **4. Overshoot** | Overshoot |
|  | Overshoot (positive sum/max, 10) |
| **5. Roughness/Irregularity** | Roughness (std, 10) |
|  | Zero-crossing rate (ZCR, 10) |
| **6. Rolling Statistics** | Rolling mean (window=5) |
|  | Rolling std (window=5) |
|  | Rolling coefficient of variation |
|  | Rolling range (window=10) |
| **7. Rate Features** | Pressure change rate |
|  | Rise rate |
|  | Fall rate |
|  | Rise/Fall ratio |
|  | Short-term max rate |
|  | Mean rate (rolling) |
| **8. Peak Shape** | Peak height |
|  | Peak width (FWHM) |
|  | Peak sharpness |
|  | Post-peak fall |
|  | Shape index |
| **9. Peak / Frequency** | Peak count (last 120 points) |
|  | FFT-dominant frequency |
| **10. Area & Slope** | Peak area (rolling sum, 10) |
|  | Peak sustain (rolling mean, 10) |
|  | Slope (window=30) |
| **11. Energy** | Short-term energy (sum diff², 10) |

ZCR, zero-crossing rate; FWHM, full width at half maximum; FFT, fast Fourier transform; std, standard deviation; diff², sum of squared differences.

**Supplementary Table S2. Performance Comparison of Machine Learning Models for IRP Artifact Detection (Entire Test Dataset)**

**Supplementary Table S3. Performance Comparison of Machine Learning Models for IRP Artifact Detection in High-Pressure Segments (IRP ≥30 mmHg)**

| **Model** | **Accuracy** | **Recall** | **Precision** | **Specificity** | **F1 score** | **ROC-AUC** |
| --- | --- | --- | --- | --- | --- | --- |
| **Random Forest** | 0.92  (0.92-0.92) | 0.81  (0.80-0.82) | 0.39  (0.38-0.39) | 0.92  (0.92-0.92) | 0.52  (0.52-0.53) | 0.95  (0.95-0.95) |
| **LightGBM** | 0.94  (0.93-0.94) | 0.70  (0.70-0.71) | 0.45  (0.44-0.46) | 0.95  (0.95-0.95) | 0.55  (0.54-0.56) | 0.95  (0.95-0.95) |
| **XGBoost** | 0.91  (0.91-0.91) | 0.81  (0.80-0.81) | 0.37  (0.36-0.38) | 0.92  (0.92-0.92) | 0.51  (0.50-0.51) | 0.95  (0.94-0.95) |

| **Model** | **Accuracy** | **Recall** | **Precision** | **Specificity** | **F1 score** | **ROC-AUC** |
| --- | --- | --- | --- | --- | --- | --- |
| **Random Forest** | 0.75  (0.74-0.76) | 0.90  (0.89-0.91) | 0.47  (0.47-0.49) | 0.71  (0.70-0.71) | 0.62  (0.61-0.63) | 0.97  (0.97-0.98) |
| **LightGBM** | 0.83  (0.82-0.83) | 0.87  (0.86-0.88) | 0.58  (0.56-0.59) | 0.81  (0.81-0.82) | 0.70  (0.69-0.71) | 0.97  (0.97-0.97) |
| **XGBoost** | 0.74  (0.73-0.75) | 0.90  (0.89-0.91) | 0.46  (0.45-0.47) | 0.69  (0.68-0.70) | 0.61  (0.59-0.62) | 0.97  (0.97-0.97) |


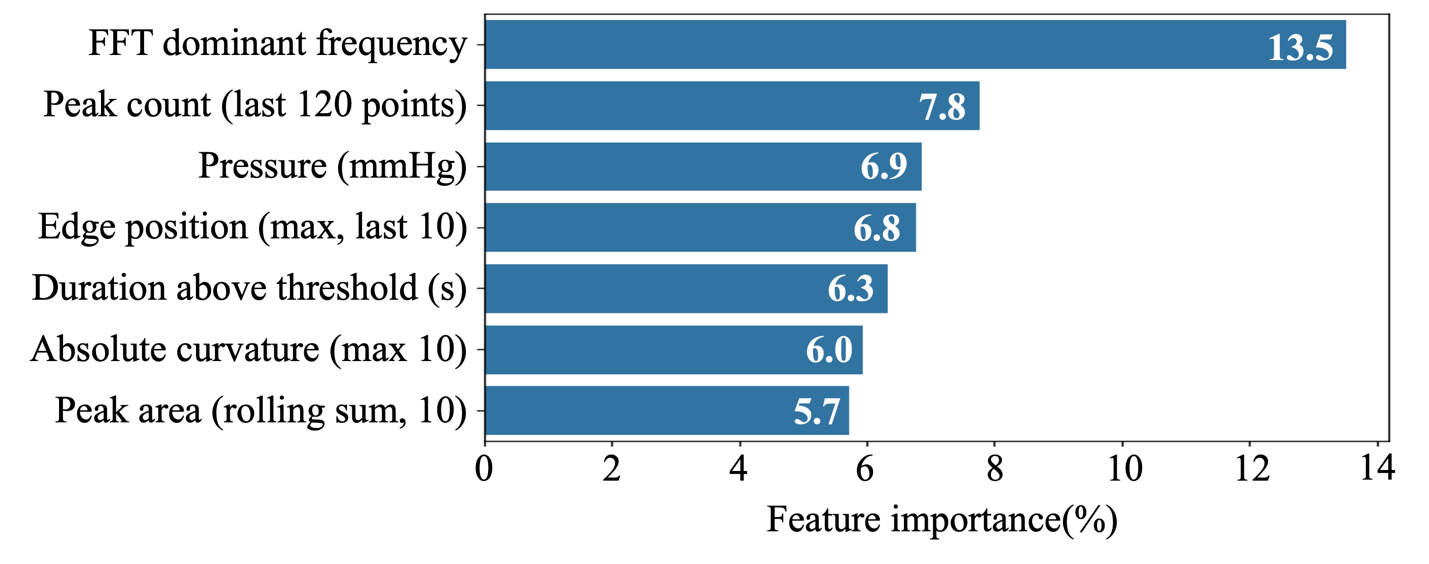
**Supplementary Fig. S1. Feature Importance for Artifact Detection from SHAP Analysis**

Horizontal bars depict the relative contribution (%) of each feature to predictions of the Light Gradient Boosting Machine (LightGBM) model as quantified by SHapley Additive exPlanations (SHAP). The Fast Fourier Transform (FFT) Dominant Frequency contributed the most to model output, followed by Peak Count (last 120 points), Absolute Pressure, Edge Position (maximum in the last 10 points), Duration Above Threshold (> 30 mmHg), Absolute Curvature (maximum in the last 10 points), and Peak Area (rolling sum over 10 points). These features together accounted for more than 50% of the model’s predictive contribution, highlighting their central role in artifact detection.
